# Impact of Cell Type and Epitope Tagging on Heterologous Expression of G Protein-Coupled Receptor: A Systematic Study on Angiotensin Type II Receptor

**DOI:** 10.1371/journal.pone.0047016

**Published:** 2012-10-08

**Authors:** Lili Jiang, Gladys M. K. Teng, Elaine Y. M. Chan, Shannon W. N. Au, Helen Wise, Susanna S. T. Lee, Wing-Tai Cheung

**Affiliations:** 1 School of Biomedical Sciences, The Chinese University of Hong Kong, Shatin, New Territories, Hong Kong, China; 2 School of Life Sciences, The Chinese University of Hong Kong, Shatin, New Territories, Hong Kong, China; Medical School of Hannover, United States of America

## Abstract

Despite heterologous expression of epitope-tagged GPCR is widely adopted for functional characterization, there is lacking of systematic analysis of the impact of expression host and epitope tag on GPCR expression. Angiotensin type II (AT2) receptor displays agonist-dependent and -independent activities, coupling to a spectrum of signaling molecules. However, consensus has not been reached on the subcellular distributions, signaling cascades and receptor-mediated actions. To examine the contributions of host cell and epitope tag on receptor expression and activity, epitope-tagged AT2 receptor variants were transiently or stably expressed in HEK293, CHO-K1 and PC12 cells. The epitope-tagged AT2 receptor variants were detected both on the cell membrane and in the perinuclear region. In transiently transfected HEK293 cells, Myc-AT2 existed predominantly as monomer. Additionally, a ladder of ubiquitinated AT2 receptor proteins was detected. By contrast, stably expressed epitope-tagged AT2 receptor variants existed as both monomer and high molecular weight complexes, and the latter was enriched in cell surface. Glycosylation promoted cell surface expression of Myc-AT2 but had no effect on AT2-GFP in HEK293 cells. In cells that stably expressed Myc-AT2, serum starvation induced apoptosis in CHO-K1 cells but not in HEK293 or PC12 cells. Instead, HEK293 and PC12 cells stably expressing Myc-AT2 exhibited partial cell cycle arrest with cells accumulating at G1 and S phases, respectively. Taken together, these results suggest that expression levels, subcellular distributions and ligand-independent constitutive activities of AT2 receptor were cell type-dependent while posttranslational processing of nascent AT2 receptor protein was modulated by epitope tag and mode of expression.

## Introduction

Heterologous expression of epitope-tagged G protein-coupled receptor (GPCR) is a common and convenient way to study the subcellular localization and cellular signaling cascades, especially for specific antibodies are lacking and/or specific ligands are not available [1], [2]. On the other hand, green fluorescent protein (GFP)-tagging has been found to alter the channel property of human acetylcholine receptor [3] and glutamate receptor [4]. Furthermore, GFP has been shown to impair the actin-myosin interaction in muscle cells [5]. However, there is lacking of systematic analysis whether the expression host and the epitope-tag exert any functional impact on GPCR expression.

The vasoactive peptide angiotensin II (ANGII) exerts its biological effects via two receptor subtypes known as angiotensin type I (AT1) and type II (AT2) receptors, which are members of the GPCR suprerfamily [6]. AT1 receptor mediates the majority of the classical biological functions of ANGII and plays an important role in regulation of blood pressure, water and electrolyte balance, thirst, hormone secretion and renal function [7]. In contrast, the AT2 receptor has involved in growth and development, wound healing and tissue injure, and pathophysiological changes in various cardiovascular diseases [8], [9]. However, due to the low expression of AT2 receptor in adult tissues and lacking of specific agonist, the pathophysiological functions of AT2 receptor are largely unknown and controversial [10]. Molecular, pharmacological and cellular studies have demonstrated that AT2 receptor displays agonist-dependent [11] and -independent [12] activities, coupling directly or indirectly to a spectrum of signaling molecules including phosphatases [13], kinases [14], G proteins [15] and Na^+^, K^+^-ATPase [16]. However, contradictory results have been reported. For instance, AT2 receptor has been found both to activate [17] and to inhibit ERK 1/2 [18].

Although anti-AT2 receptor antibodies are currently available either commercially or from individual research groups, there are controversy whether the antibodies can be used to detect endogenously expressed AT2 receptors [19]. In order to examine and to compare the effects of species and cell-type specificities on receptor expression and cellular functions, rat AT2 receptor tagged C-terminally with GFP (AT2-GFP) or FLAG (AT2-FLAG); and N-terminally with Myc (Myc-AT2) or HA (HA-AT2) were transiently or stably expressed in three cell lines including human embryonic kidney HEK293, rat pheochromocytoma PC12 and Chinese hamster ovary CHO-K1 cells. Different epitope-tagged AT2 receptor variants displayed similar subcellular distributions in HEK293 cells, but cell surface expression of Myc-AT2 receptor variant was promoted by glycosylation but not AT2-GFP receptor variant. Myc-AT2 receptor variant expression induced partial cell-cycle arrest in HEK293 and PC12 cells but had no effect in CHO cells. These results suggest that expression, posttranslation processing, cellular compartmentation and ligand-independent AT2-mediated cellular activity are specifically affected by the host cells as well as the epitope tag.

## Materials and Methods

### Antibodies

Mouse monoclonal anti-GFP antibody (Cat. 632381, Clontech), mouse monoclonal anti-HA antibody (Cat. Sc-7392, Santa Cruz) and mouse monoclonal anti-FLAG M2 antibody (Cat. F3165, Sigma-Aldrich) were used for immunofluorescent staining and western blot. Rabbit polyclonal anti-GFP antibody (Cat. 632460, Clontech) and rabbit polyclonal anti-FLAG antibody (Cat. F7425, Sigma-Aldrich) were used for immunoprecipitation. Mouse monoclonal anti-Myc antibody (Cat. sc-40, Santa Cruz) and monoclonal anti-ubiquitin (Cat. Sc8017, Santa Cruz) was used for immunofluorescent staining, immuoprecipitation and western blot.

### Angiotensin At2 Expression Constructs

Full-length rat *At2* cDNA was PCR-amplified with the pBluescript II KS (+)/AT2 construct as template [11] using primer pairs containing an EcoR I and Xho I in the forward and reverse primer, respectively (Table 1). To generate AT2-FLAG, Myc-AT2 and HA-AT2 expression constructs, the *At2* PCR fragment was then subcloned into EcoR I- and Xho I-linearized pCMV-tag4A (Stratagene), pCMV-Myc (Clontech) and pCMV-HA (Clontech) vectors, respectively, as described previously [20]. For AT2-GFP receptor variant, in order to minimize any possible steric hindrance arose from the bulky EGFP protein that blocks the interactions between the C-terminal tail of AT2 receptor and the intracellular signaling proteins, a 45 oligonuclotides encoding a Gly_10_Ser_5_ (SGGGGSGSGSSGGGG) peptide linker was inserted between the AT2 and EGFP genes using overlapping PCR. Following enzyme restriction, the AT2-Gly_10_Ser_5_-EGFP amplicons were then subcloned into the EcoR I – Xba I sites of pEGFP-N1 (Clontech). For generating mammalian Myc-AT2 expression construction, the Xba I – Xba I Myc-AT2 fragment of pCMV-Myc/AT2 was subcloned into a pCDNA3 (Invitrogen) vector at the Xba I restriction site. All *At2* expression constructs were sequenced and confirmed by Beijing Genomics Institute Hong Kong (BGI-HK).

**Table 1:** PCR primers for rat *At2* cDNA sublconing.

Constructs		Primers	Nucleotide sequences
AT2-GFP	AT2-linker	Forward	5′-CCG GAA TTC CGG ATG AAG GAC AAC TTC AGT-3′
			*EcoR I*
		Reverse	5′-AGA ACC GCT GCC TGA ACC GCC TCC ACC ACT
			TGG ATC CGC GGC AGA CAC AA-3′
			*Bam HI*
	Linker-GFP	Forward	5′-TCA GGC AGC GGT TCT AGC GGC GGT GGC
			GGA CCG GTC GCC ACC ATG GTG AG-3′
			*Age I*
		Reverse	5′-TGA TCT AGA GTC GCG GCC GCT TTA CTT GTA-3′
			*Xbal I Not I*
AT2-FLAG		Forward	5′-CCG GAA TTC CGG ATG AAG GAC AAC TTC AGT-3′
			*EcoR I*
		Reverse	5′-CCG CTC GAG CGG AGA CAC AAA GGT GTC CAT-3′
			*Xho I*
Myc-AT2/HA-AT2		Forward	5′-CCG GAA TTC AGC GGA TGA AGG ACA ACT TCA GT-3′
			*EcoR I*
		Reverse	5′-CCG CTC GAG TTA AGA CAC AAA GGT GTC CAT-3′
			*Xho I*

### Cell Culture and Drug Treatment

Cell lines were obtained from the American Type Culture Collection (ATCC), maintained with penicillin and streptomycin-supplemented medium and kept in a humidified incubator at 37°C with 5% CO_2_. Fetal bovine serum (FBS) and culture medium were purchased from Invitrogen. Chinese hamster ovary cells (CHO-K1) were maintained in F12 medium supplemented with 10% FBS; human embryonic kidney cells (HEK293) were maintained in DMEM medium supplemented with 10% FBS; rat pheochromocytoma cells (PC12) were cultured in F12-K medium containing 2.5% FBS and 15% horse serum. For cell proliferation assay, cells were cultured in the absence or presence of 1 µM ANGII or 10 µM selective AT2 non-peptide antagonist PD 123319 (Sigma-Aldrich) as indicated. For deglycosylation treatment, cells were cultured in the presence of 1 µg/ml tunicamycin (Sigma-Aldrich) for 24 hr before cell lysis. For cell surface immunoprecipitation, cells were cultured in serum free medium at 37°C overnight.

### Transient and Stable AT2 Expression

Cells were transiently transfected with *At2* expression construct (1 µg DNA per well for 6-well dish; 5∼10 µg DNA per 100 mm dish) using Lipofectamine 2000 (Invitrogen) according to the manufacturer’s instructions. Cells that stably expressed AT2 receptor were selected against 1 mg/ml G418 (Chemsonic) and clonal cell lines were obtained by limiting dilution in 96-well plates. Expression of AT2 receptor in clonal cell lines was examined by immunofluorescent staining.

**Figure 1:**
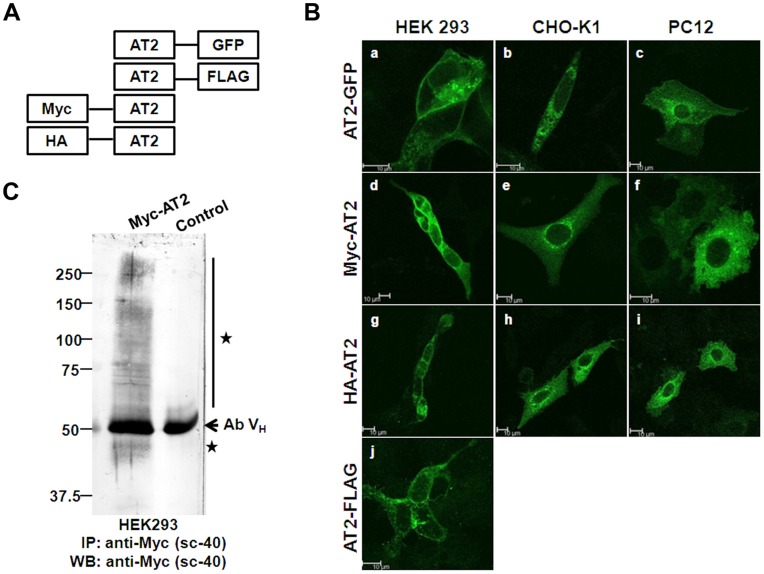
Transient expressions of epitope-tagged AT2 receptors. (A) Schematic illustration of C-terminally or N-terminally tagged AT2 receptor variants. (B) Confocal images of transiently expressed epitope-tagged AT2 receptor variants. Plasmid (1 µg) was transiently transfected into cells (on a 25-mm coverslip). After 24 hr, cells that transfected with AT2-GFP were examined by live cell imaging; cells that transfected with Myc-AT2, HA-AT2 or AT2-FLAG were fixed, permeabilized and probed with an epitope-specific antibody as described in Methods. Following incubation with an FITC-conjugated secondary antibody, fluorescent images of transiently transfected cells were captured using a 63× water immersion objective with a Leica SP5 confocal microscope. (C) Pull-down immunoblot protein analysis of HEK293 cells that were transiently transfected with Myc-AT2. The Myc-AT2 expression construct or control empty vector (5 µg) was transiently transfected into HEK293 cells (in a 100-mm dish) as described in Methods. After 48 hr, cells were lysed, immunoprecipitated and probed with an anti-Myc antibody. Specific immunoreactive protein bands are indicated with asterisks. Ab V_H_: Antibody heavy chain. Data shown is a representative of 2–3 independent experiments with similar results.

### Immunofluorescent Staining and Confocal Microscopy

Immunofluorescent staining was performed as described previously with minor modifications [21]. Cells were seeded and grown on coverslip for 48 hr. For HEK293 cells, the coverslips were pre-treated with 0.01% (w/v) poly-L-lysine for 15 min. The cells were washed in PBS and fixed with absolute methanol at −20°C for 10 min. Following fixation, the cells were washed with a Tris buffer (50 mM Tris-HCl, pH 7.4). The non-specific binding sites were blocked with a blocking buffer (50 mM Tris-HCl, pH 7.4, 0.5% Triton X-100, 10% goat serum) for 30 min and then washed with a Tris/Triton buffer (50 mM Tris-HCl, pH 7.4, 0.1% Triton X-100). Next, the cells were incubated with an epitope-specific primary antibody (1 µg in 200 µl) at 4°C overnight. Following washing with the Tris/Triton buffer, cells were incubated with an FITC-conjugated secondary antibody (Invitrogen, 1 µg in 200 µl) for 1 hr at room temperature. The coverslips were washed and mounted on glass slides with an anti-fade mounting medium (0.1 M Tris-HCl, pH 8.0, 90% glycerol, 0.02% sodium azide, 2.3% 1, 4-diazabicyclo-[2,2,2]-octane). Images were captured using a 63× water immersable objective with a Leica SP5 confocal microscope. Confocal images were further analyzed and processed with Leica LAS AF software and Photoshop (Adobe). Data were collected from 2∼3 independent experiments, and 2–5 slides were examined in each experiment.

**Figure 2:**
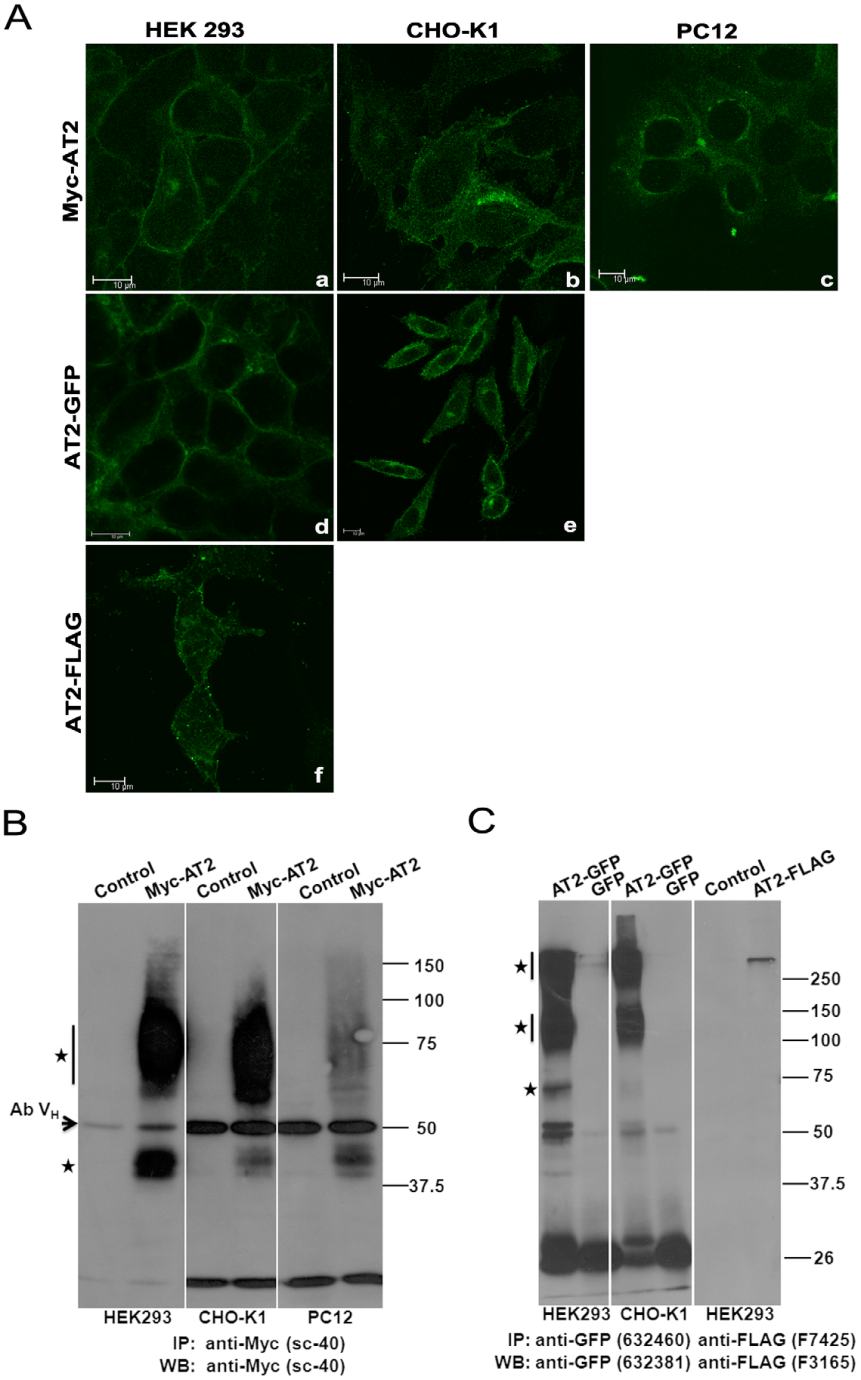
Stable expressions of epitope-tagged AT2 receptors. (A) Confocal images of stably expressed C-terminally or N-terminally epitope-tagged AT2 receptor variants. Stably transfected cells were seeded and grown on coverslip for two days. Following fixation and permeabilization, the cells were probed with epitope-specific antibody and then incubated with an FITC-conjugated secondary antibody as described in Methods. Fluorescent images were captured using a 63× water immersion objective with a Leica SP5 confocal microscope. Pull-down immunoblot protein analysis of cells stably transfected with N-terminally tagged (B) or C-terminally tagged (C) AT2 receptor variants. To increase surface expression of AT2, CHO-K1 and PC12 cells that stably expressed Myc-AT2 were serum-starved overnight. Stably transfected cells or respective controls were lysed in RIPA buffer, immunoprecipitated and probed with an anti-Myc (B), anti-GFP or anti-FLAG (C) antibody as indicated. Cells stably transfected with corresponding empty vector were used as controls. Specific immunoreactive protein bands are indicated with asterisks. Ab V_H_: Antibody heavy chain. Data shown is a representative of 2–3 independent experiments with similar results.

### Immunoprecipitation and Western Blot

Cells were lysed in RIPA buffer [0.1 M Tris-HCl pH 7.4, 150 mM NaCl, 1% sodium deoxycholate, 1% Triton X-100, 5 mM EDTA supplemented with protease inhibitor cocktail (Roche)] and clarified by centrifugation at 10,000 g for 10 min at 4°C. Protein concentration was determined using bicinchoninic acid (BCA) protein assay kit (Pierce) according to the manufacturer’s instructions. Supernatant (∼1 mg protein in 1 ml) was incubated with primary antibody (2 µg) and 50 µl of protein A or protein G agarose (Roche) at 4°C overnight with gentle shaking. After washing, the pellets were resuspended in SDS sample buffer (2% SDS, 10% glycerol, 40 µl/ml β-mercaptoethanol and 0.01% bromphenol blue in 50 mM Tris-HCl, pH 6.8) and immunoprecipitated proteins were separated in 10% SDS-PAGE under reducing conditions and then electro-transferred to nitrocellulose membranes (Whatman). The membrane was blocked in an immunobloting buffer (25 mM Tris-HCl; pH7.4, 1 mM CaCl_2_, 40 mM NaCl, 0.1% NP40 and 5% milk) for 1 hr and then incubated with corresponding primary antibody at 4°C overnight. Following incubation with horseradish peroxidase-conjugated secondary antibodies (1∶1000 dilution, GE Healthcare), immunoreactive protein bands were visualized using ECL reagent (GE Healthcare). The immunoreative bands were digitalized using a flat-bed scanner (EPSON GT9500) at 300 dpi and quantified using UN-SCAN-IT software (Silk Scientific). Band intensity was presented as total pixel value with a 256 grey scale for each pixel. Blank area was used as background control. Data was normalized against the controls. Differences between normalized data were analyzed with GraphPad Prisms 5 (GraphPad Software).

**Figure 3:**
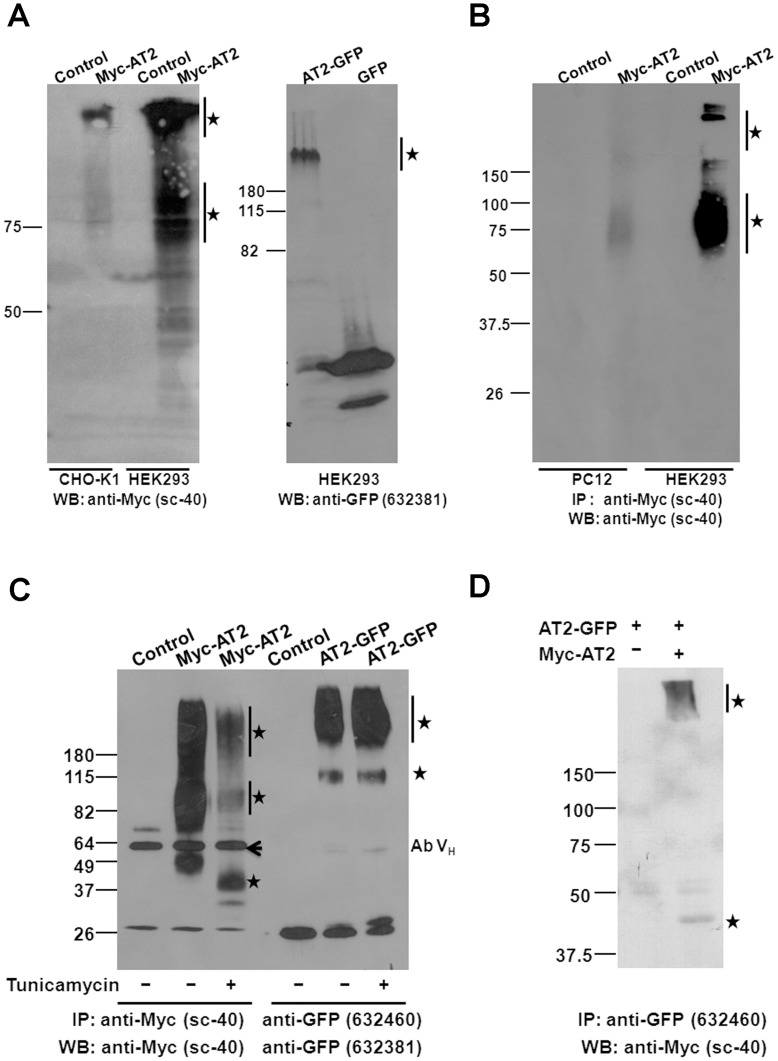
Roles of post-translational processing on cell surface expression of AT2 receptors. (A) Western protein blot analysis of the membrane fraction of cells that stably transfected with epitope-tagged AT2 receptor variants. Cells that stably expressed Myc-AT2 were cultured in 100 mm dish until confluence. Cells were then cultured in serum-free medium overnight the day before cell lysis. Membrane fraction was prepared as described in Methods. Protein blot was probed with an anti-GFP or an anti-Myc antibody as indicated. (B) Cell surface immunoprecipitation of Myc-AT2. Cells were cultured until confluence and then incubated with serum-free medium overnight the day before cell lysis. Cell surface AT2 receptors were immunoprecipitated as described in Methods. Immunoprecipitated proteins were separated in 10% SDS-PAGE and protein blot was probed with an anti-Myc antibody. (C) Glycosylation on epitope-tagged AT2 receptor variants. HEK293 cells (100 mm disk with >90% confluence) that stably expressed Myc-AT2 or AT2-GFP were treated with or without 1 µg/ml tunicamycin for 24 hr. Cells were then lysed in RIPA buffer, epitope-tagged AT2 is immunoprecipitated, and protein blot was probed with an anti-Myc or an anti-GFP antibody as indicated. (D) Dimer and oligomer formation of AT2 receptors. The AT2-GFP expression construct (5 µg) was transiently co-transfected with Myc-AT2 expression construct (5 µg) or empty vector controls into HEK293 cells. Two days later, cells were lysed in the RIPA buffer. The dimeric or oligomeric AT2 receptors were immunoprecipitated with a polyclonal anti-GFP antibody and detected with a monoclonal anti-Myc antibody. Cells stably transfected with corresponding empty vector were used as controls. Specific immunoreactive protein bands are indicated with asterisks. Ab V_H_: Antibody heavy chain. Data shown is a representative of 2–4 independent experiments with similar results.

**Figure 4:**
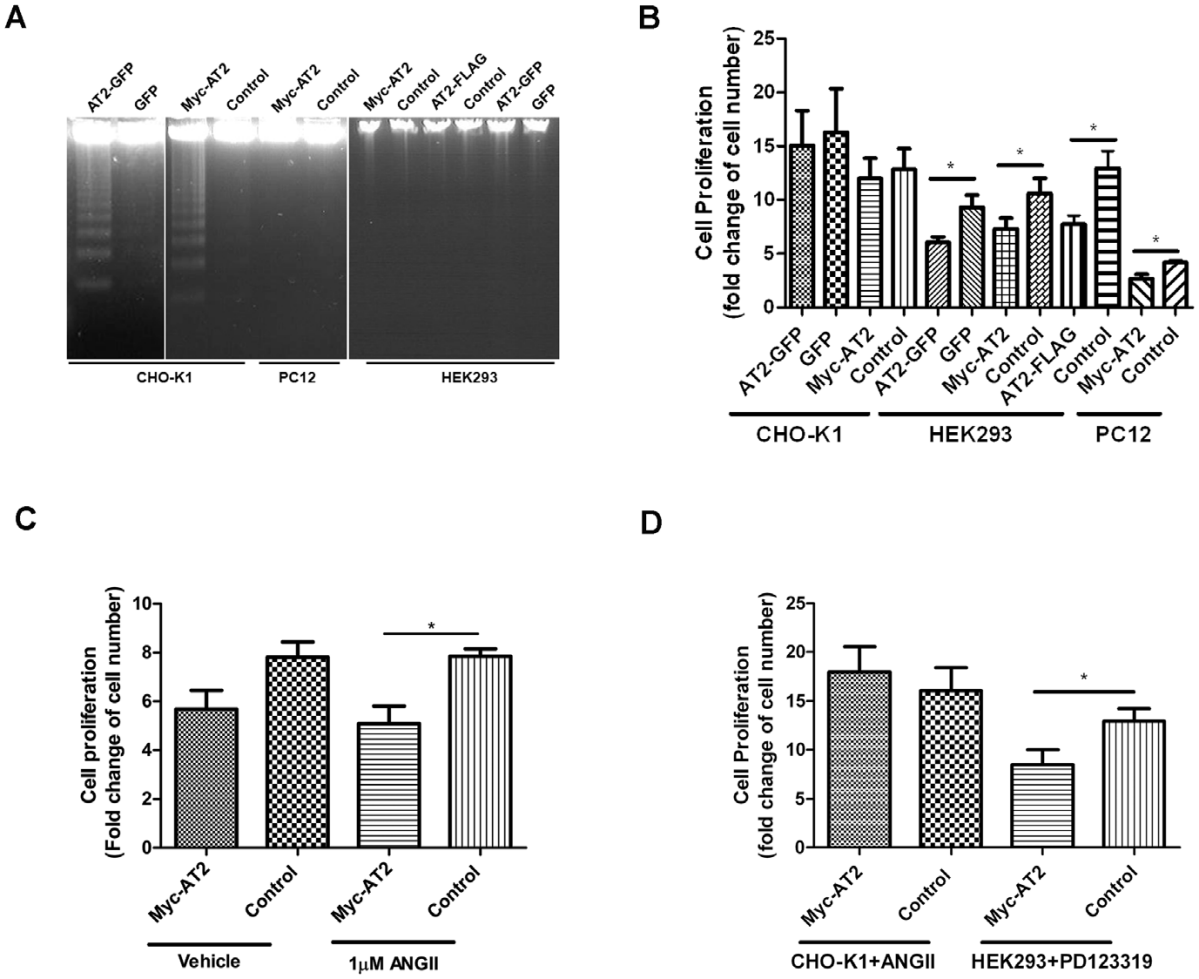
Ligand-independent constitutive activities of epitope-tagged AT2 receptors. (A) Serum starvation-induced DNA fragmentation. Stably transfected CHO cells (5×10^5^), PC12 cells (5×10^5^), and HEK cells (1×10^6^) were cultured in serum-free medium for 72 hr. Genomic DNA was extracted and fractionated on 2% TAE agarose gel as described in Methods. (B) Cell proliferation analysis. Stably transfected CHO cells (1×10^5^ cells), PC12 cells (5×10^5^ cells) and HEK293 cells (5×10^5^ cells) were seeded on 60-mm dishes and cultured for 5 days. (C) Stably transfected HEK293 cells (5×10^5^ cells on 60 mm dish) were cultured for 5 days in the absence or presence of 1 µM ANGII. (D) Stably transfected CHO-K1 (1×10^5^ cells on 60 mm dish) and HEK293 cells (5×10^5^ cells on 60 mm dish) were cultured for 5 days in the presence of 1 µM ANGII or 10 µM PD 123319, respectively. Cell numbers were counted using Beckman coulter Vi-cell TM XR cell viability analyzer. Cell proliferation were expressed as fold change of cell number. Data shown is mean ± SEM from 4∼6 independent experiments. *indicates *P*<0.05.

For ubiquitination assay, 48 hr after transfection with pHM6/HA-ubiquitin plasmid (5 µg), transfected cells were incubated with proteasome inhibitor MG132 (10 µg/ml, Sigma-Aldrich) for 5 hr prior lysing in 1 ml of RIPA buffer containing 20 mM N-ethylmaleimide (Sigma-Aldrich).

### Subcellular Fractionation

Cell membrane fraction was prepared as described by Nahmias and coworkers [22] with minor modifications. Cells were washed in PBS, resuspended in 1 ml of an extraction buffer (20 mM Tris-HCl; pH 7.5, 2 mM EDTA, 2 mM PMSF supplemented with protease inhibitor cocktail) and incubated on ice for 15 min. The cells were then lyzed by incubating consecutively in liquid nitrogen and 37°C water bath for 5 cycles. Cell lysate was centrifuged at 1,500 g for 8 min to remove remaining cell debris. The supernatant was further centrifuged at 100,000 g for 1 hr at 4°C. The resulting membrane pellet was dissolved in SDS sample buffer, membrane proteins were separated in 10% SDS-PAGE and immunoblotted with indicated antibodies.

**Table 2:** Cell cycle profile of stable cell lines expressing epitope-tagged AT2 receptor variants.

	HEK293						PC12	
	AT2-FLAG		AT2-GFP		Myc-AT2		Myc-AT2	
	Control	Experiment	Control	Experiment	Control	Experiment	Control	Experiment
G1	46.89±1.45	55.78±0.94***	50.45±2.62	57.98±1.70*	46.43±1.90	52.06±1.63*	67.77±0.85	58.36±0.57
S	42.28±1.32	38.10±1.46	42.82±2.89	34.21±1.44	45.58±1.31	39.40±1.06	18.34±0.18	25.14±0.80**
G2/M	9.68±1.84	7.27±1.08	6.69±0.97	7.85±0.89	8.00±1.00	8.54±0.88	13.89±0.92	16.50±1.17

Stably transfected HEK cells (1×10^6^) and PC12 cells (5×10^5^) were seeded in 60 mm dish. After 2 days, the cells were collected and cell cycle analysis was performed as described in Methods. Cells stably transfected with empty vector were used as Control. Distributions of cells in each phase of a cell cycle were expressed as percentage of total cell analyzed. Data shown is mean ± SEM of at least 3 independent experiments. Differences between means were compared with unpaired Student’s *t* test using GraphPad Prism 5.

* *P*<0.05;

** *P*<0.01;

*** *P*<0.001.

### Cell Surface Immunoprecipitation

Cell surface immunoprecipitation was performed as described by Michineau and co-workers [23]. The whole process was conducted at 4°C in the presence of 10 mM iodoacetamide. HEK293 cells stably expressing Myc-AT2 were washed in PBS, incubated with 2 ml of a blocking buffer [0.2% (w/v) BSA in PBS] for 1 hr and then with an anti-Myc antibody (4 µg in 2 ml of blocking buffer) for 2 hr. After two washes in blocking buffer and two washes in PBS, the cells were lysed in 1 ml of RIPA buffer and incubated with 50 µl of protein G agarose overnight at 4°C with gentle shaking.

### DNA Fragmentation

DNA fragmentation indexing the cellular apoptotic response was measured as described by Zhu and Wang [24] with minor modifications. Briefly, cells were cultured in serum-free medium for 72 hr, then lysed in 400 µl of a DNA lysis buffer (200 mM Tris-HCl, pH 8.3, 100 mM EDTA and 1% SDS, supplemented with 0.25 mg/ml proteinase K) and incubated at 50°C for 2 hr. The cell lysate was mixed with 150 µl of saturated NaCl solution, vortexed vigorously and centrifuged at 6500 g for 15 min. Supernatant was mixed with 1 ml of ice cold absolute ethanol and centrifuged at 15,000 g for 20 min. DNA pellet was washed with 75% ethanol, air dried, dissolved in RNase A solution (0.2 mg/ml) and incubated at 37°C water bath for 90 min. DNA was separated in a 2% TAE agarose gel and visualized under UV illumination.

**Figure 5:**
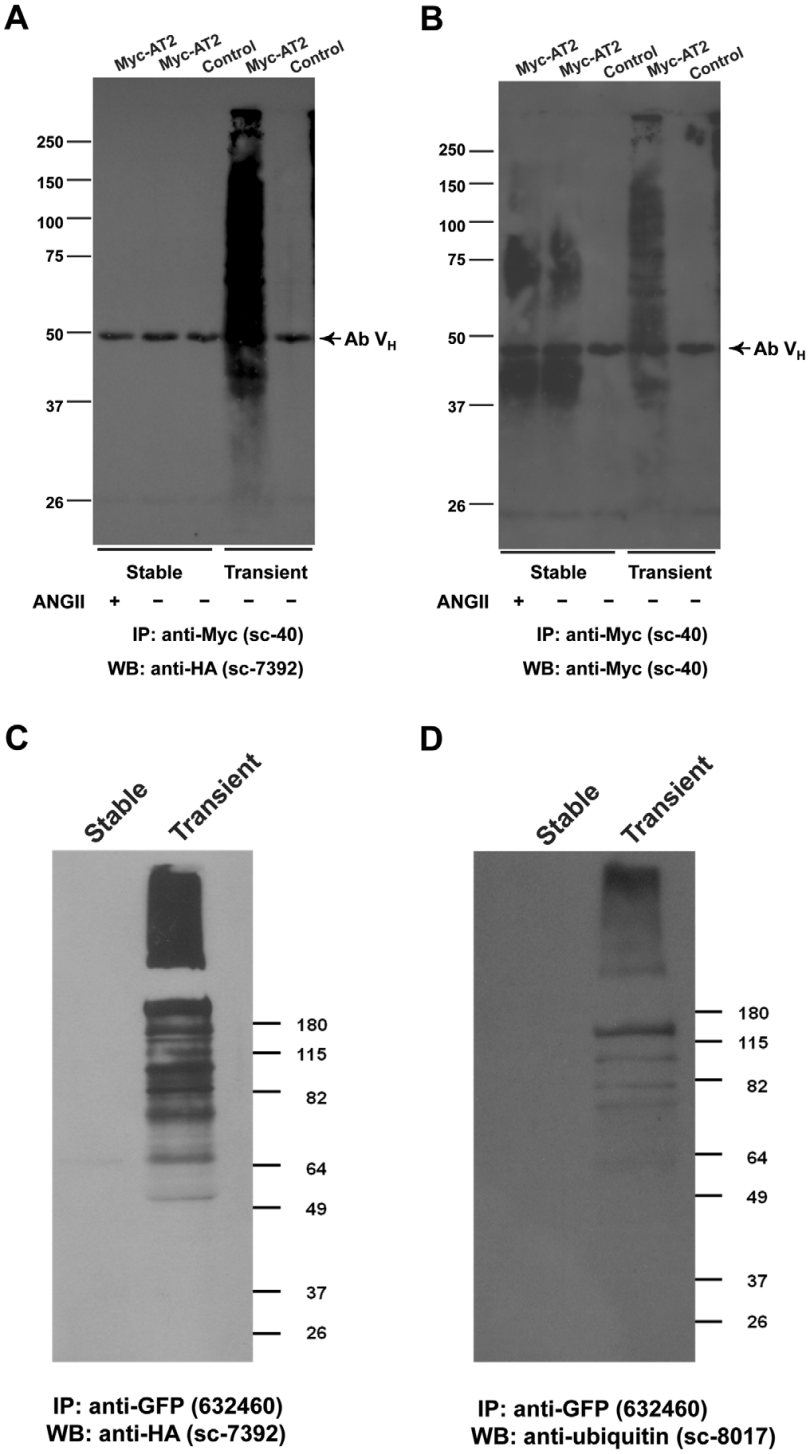
Ubiquitination of transiently expressed epitope-tagged AT2 receptors. (A, B) The HA-ubiquitin expression construct (5 µg) were transiently co-transfected with Myc-AT2 expression construct or control empty vector (5 µg) into HEK293 cells (in a 100-mm dish); or HA-ubiquitin expression construct (5 µg) was transiently transfected into HEK293 cells (in 100-mm dish) that stably expressed Myc-AT2 or control HEK293 cells that stably transfected with empty vector. After 48 hr, cells were incubated with 10 µg/ml MG132 for 5 hr and then lysed in 1 ml of RIPA buffer containing 20 mM N-ethylmaleimide. Cell lysate was subject to immunoprecipitation with an anti-Myc antibody and then probed with an (A) anti-HA or (B) anti-Myc antibody. (C, D) Expression constructs of HA-ubiquitin (5 µg) and AT2-GFP (5 µg) were transiently co-transfected into HEK293 cells (in 100 mm dish); or HA-ubiquitin expression construct (5 µg) was transiently transfected into HEK293 cells that stably expressed AT2-GFP (in a 100-mm dish). After 48 hr, cells were lysed in 1 ml of RIPA buffer. Cell lysate was subject to immunoprecipitation with an anti-GFP antibody and then probed with an (C) anti-HA or (D) anti-ubiquitin antibody. Ab V_H_: Antibody heavy chain. Data shown is representative of 2–3 independent experiments with similar results.

### Cell Proliferation and Cell Cycle Analyses

Cell proliferation and cell cycle analysis were performed as previously described [25]. For cell proliferation analysis, transfected CHO-K1 cells (1×10^5^ cells), PC12 cells (5×10^5^ cells) and HEK 293 cells (5×10^5^ cells) were seeded on 60-mm dishes and cultured for 5 days at 37°C. Cells were then trypsinized and resuspended in cell culture medium. Aliquots of suspended cells were then subject to viability and cell counting using Vi-cell™ XR cell viability analyzer (Beckman counter). Cell proliferation was expressed as fold change of cell number after 5-day culture. Data is expressed as mean ± SEM of 4∼6 independent experiments and analyzed with GraphPad Prism 5.

**Table 3:** Characteristics and ligand-independent activities of AT2 receptor variants expressed in different cell types.

Characteristics and ligand-independent cellular activity		HEK293			PC12			CHO-K1		
		Myc-AT2	AT2-GFP	AT2-FLAG	Myc-AT2	AT2-GFP	AT2-FLAG	Myc-AT2	AT2-GFP	AT2-FLAG
**Transient**	**Molecular Mass**	Multiple Bands	*	*	*	*	*	*	*	*
	**Ubiquitinylation**	+	+	*	*	*	*	*	*	*
**Stable**	**Molecular Mass (kDa)**	45, 65-100	65-70, 110-140,>250	>250	45, 65-100	Stable cell line was unable to establish	Stable cell line was unable to establish	45, 65-100	65-70, 110-140, >250	Stable cell line was unable to establish
	**Ubiquitinylation**	–	–	*	*			*	*	
	**Glycosylation**	+	–	+	+			+	–	
	**DNA fragmentation**	–	–	–	–			+	+	
	**Proliferation**	↓	↓	↓	↓			–	–	
	**Cell cycle arrest**	G1	G1	G1	S			–	–	

The + symbol indicates the cellular activity is present, while the – symbol indicates its absence. The ↓ symbol indicates the cellular activity is decreased, while the – symbol indicates no change. The * symbol indicates the activity was not determined or not detectable.

For cell cycle analysis, suspended cells were fixed in 70% ethanol at 4°C overnight and subsequently resuspended in PBS containing RNase A (8 µg/ml) and propidium iodide (PI; 40 µg/ml) (Sigma) for 15 min. The stained cells were filtered through a 53-µm Spectra/Mesh® polyester filter (Spectrum Laboratories) to remove cell clamps and then cells were subject to flow cytometrical analysis with FACS Canto (BD Biosciences). Data is expressed as mean ± SEM of at least 3 independent experiments and analyzed with GraphPad Prism 5.

### Statistical Analysis

Data is expressed as mean ± SEM of 3∼6 independent experiments. Differences between means were evaluated with an unpaired Student’s *t* test using GraphPad Prism (version 5), and a *P* value of equal or smaller than 0.05 is indicated as statistically significant difference.

## Results

### Transient Expression of Various N- or C-terminally Tagged AT2 Receptor Variants

To examine the expression and cellular distribution, AT2 receptor variants were constructed such that the rat AT2 receptor was tagged at the C-terminus with GFP or FLAG, or at the N-terminus with Myc or HA (Figure 1A). Transient transfection was initially performed in HEK293, PC12 and CHO-K1 cells. Confocal microscopy study indicated expressions of AT2 receptor variants were critically affected by the expression host. The AT2 receptor variants were mainly expressed on cell membranes and in the cytosol in HEK293 cells (Figure 1B, panels a, d, g, j). By contrast, the AT2 receptor variants were abundantly expressed in the perinuclear region in CHO-K1 (Figure 1B, panels b, e, h) and PC12 (Figure 1B, panels c, f, i) cells, suggesting that the receptors were localized in the endoplasmic reticulum (ER) and Golgi body. Among the three cell lines, HEK293 cells displayed the highest transfection efficiency while PC12 cells were the lowest (data not shown).

Immunoprecipitation analysis of transiently transfected HEK293 cells indicated that Myc-AT2 receptor variant was expressed as monomer characterized with a predicated molecular mass of 45 kDa (Figure 1C). In addition, a ladder of high molecular weight (MW) immunoreactive bands was also detected, suggesting that the newly synthesized AT2 receptor was post-translationally modified and/or underwent oligomer formation (Figure 1C). No specific proteins were pulled down in HEK293 cells that transiently transfected with AT2-GFP, AT2-FLAG or HA-AT2 receptor variants (data not shown), suggesting that those receptor variants had a relatively lower expression efficiency than Myc-AT2.

### Stable Expression of Various N- or C-terminally Tagged AT2 Receptor Variants

To compare with the transient expressions, stable cell lines expressing various AT2 receptor variants were constructed. For each of the epitope-tagged receptor variants, 2–3 stable cell clones were obtained (Table S1). Preliminary study indicated that stable clones of an epitope-tagged AT2 receptor variant showed similar characteristics, and therefore the highest expression clone was used for subsequent experiments. Consistent with transient transfection (Table S2), Myc-AT2, AT2-GFP and AT2-FLAG receptor variants all displayed clear membrane expression in HEK293 cells (Figure 2A, panels a, d, f), but predominantly enriched in the perinuclear region in CHO-K1 and PC12 cells (Figure 2A). However, membrane expressions of AT2-GFP and Myc-AT2 receptor variants were also found in CHO-K1 (Figure 2A, panel e) and PC12 (Figure 2A, panel c) cells, respectively.

In contrast to a ladder of immunoreactive protein bands was detected in transient expression (Figure 1C), two immunoreactive protein bands were found in HEK293, CHO-K1 and PC12 cell lines that stably expressed Myc-AT2 receptor variant (Figure 2B). Protein band of 45 kDa was likely to be the Myc-AT2 monomer. However, nature of the 65∼100 kDa protein bands was not defined, the high molecular mass could be resulted from posttranslational modifications such as glycosylation and/or oligomerization of Myc-AT2 protein [12], [26].

Similar to the expression of Myc-AT2 receptor variant, three immunoreactive protein bands characterized with molecular mass of 65∼70 kDa, 110∼140 kDa and >250 kDa were pulled down in HEK293 and CHO-K1 cells that stably expressed AT2-GFP receptor variant (Figure 2C). The 65∼70 kDa protein band was close to the predicted MW of monomeric AT2-GFP protein. Those high MW immunoreactive bands could be posttranslationally modified or oligomeric AT2-GFP receptor variant. Intriguingly, HEK293 cells that stably transfected with AT2-FLAG receptor variant only showed a very high MW (>250 kDa) immunoreactive protein band (Figure 2C).

Comparing the intensities of immunoprecipitated protein bands, it is of interest to note that the expression levels of Myc-AT2 receptor variant varied among different expression hosts. The HEK293 cells gave the highest expression of Myc-AT2 receptor variant while the PC12 cells exhibited the lowest expression level (Figure 2B). Although Myc-AT2 and AT2-GFP receptor variants expressed abundantly in HEK293 cells, the expression levels of AT2-FLAG receptor variant is significantly lower (Figure 2B and C). By contrast, expression levels of AT2-GFP receptor variant were similar in both HEK293 and CHO-K1 cells (Figure 2B). These results suggested that expression level of AT2 receptor was affected both by cell type and epitope tag.

### High Molecular Weight AT2 Receptors were Enriched in the Cell Surface and Existed as Oligomeric Complex

With the detection of monomeric and high MW AT2 receptors in stably transfected cells, next we would like to examine which form was located in the cell membrane. Surface expression of AT2 receptor in stably transfected cells was enhanced by overnight serum-starvation [12], membrane fractions of stably transfected cells were isolated. Expressions of epitope-tagged AT2 receptor variants in the membrane fractions were examined by western protein analysis. Of interest, the high MW forms of Myc-AT2 and AT2-GFP receptor variants were enriched in the membrane fraction in both CHO-K1 and HEK293 cells (Figure 3A). By contrast, the putative monomeric low MW tagged AT2 receptor was barely detectable, if any (Figure 3A).

To confirm high MW AT2 receptor was enriched in cell surface, N-terminally Myc-tagged AT2 receptor was pulled down directly from the cell surface by incubating the live cells with an anti-Myc antibody. Consistent with the result of subcellular fractionation, the Myc-AT2 receptor protein in cell surface was found mainly to be high MW (65∼100 kDa) proteins in both PC12 and HEK293 cells that stably expressed Myc-AT2 receptor variant (Figure 3B).

To examine whether the high MW Myc-AT2 receptor protein underwent extensive glycosylation, stably transfected cells were cultured in the presence of tunicamycin which inhibits a series of enzymes involved in synthesis of N-linked glycoproteins [27] and is commonly used to block glycosylation of proteins [28]. Comparing to non-treated cells, the high MW protein bands of Myc-AT2 receptor variant decreased dramatically after tunicamycin treatment in stably transfected HEK293 (Figure 3C), CHO-K1 and PC12 (Figure S1) cells. In addition, the 45 kDa band also shifted to about 34 kDa, which was reported to be the non-modified MW of monomeric AT2 receptor [26].

Similarly, despite only a high MW protein band (>200 kDa) of AT2-FLAG was detected in stably transfected HEK293 cells, a protein band with a molecular mass of 75 KDa was found in addition to the high MW protein band upon tumicamycin treatment (Figure S1). The result suggested that AT2-FLAG was subject to glycosylation in HEK293 cells, but the nature of the 75 KDa protein band was unknown, and it could be homodimer of non-glycosylated AT2-FLAG receptor protein. To our surprise, tunicamycin treatment did not exert any effect on the expression of AT2-GFP receptor variant in stably transfected HEK293 cells (Figure 3C) and CHO-K1 cells (Figure S1), suggesting that the AT2-GFP receptor variant was possibly not glycosylated.

To determine whether the high MW (>200 kDa) immunoreactive band constituted of oligomeric AT2 receptors, AT2-GFP and Myc-AT2 receptor variants were transiently co-transfected into HEK293 cells. Following immunoprecipitaton with an anti-GFP antibody, monomeric form of Myc-AT2 receptor variant at 45 kDa was detected in the immunocomplex with an anti-Myc antibody (Figure 3D). In addition, immunoreactive proteins characterized with very high MW (>200 kDa) were also detected (Figure 3D). These results suggest that AT2 receptors formed homo-oligomers which were then transferred to the cell surface.

### Ligand-independent AT2-mediated Cellular Responses were Cell-type Dependent

Expression studies indicated that the host cells determined the subcellular distributions while the epitope tags affected the post-translation processing of AT2 receptors. Hence, it is of interest to examine whether receptor activity would also be modulated by host cells or epitope tags. To examine AT2 receptor activity, cells that stably transfected with various epitope-tagged AT2 receptors were compared on serum starvation-induced DNA fragmentation [29] and on constitutive activity of oligomeric AT2 receptor [12]. The CHO-K1 cells stably transfected with AT2-GFP or Myc-AT2 receptor variants exhibited DNA fragmentation after 3 days of serum-starvation while lacking of any change in control cells that transfected with empty vector, suggesting CHO-K1 cells that stably expressed AT2 receptor underwent apoptotic responses upon serum starvation (Figure 4A). However, serum-starvation did not induce any noticeable apoptotic response in PC12 and HEK293 cells stably transfected with epitope-tagged AT2 receptor variants, even after 4∼5 days of serum starvation (Figure 4A).

To investigate the constitutive activity of AT2 receptor, proliferation rates of stably transfected cell clones were examined. In comparison with empty vector transfected control cells, HEK293 and PC12 cells that stably expressed Myc-AT2 or AT2-GFP receptor variants displayed a 31–36% reduction in cell growth, but had no effect on the growth of CHO-K1 cells (Figure 4B). The addition of 1 µM ANGII did not further enhanced the AT2-mediated growth inhibition in HEK 293 cells that stably expressed Myc-AT2 receptor variant (Figure 4C). On the other hand, the addition of 10 µM PD123319, an AT2 antagonist, also did not reverse the AT2-mediated growth inhibition in HEK293 cells that stably expressed Myc-AT2 receptor variant (Figure 4D). Similarly, the addition of 1 µM ANGII in culture medium did not exert any inhibitory effect on the growth of CHO-K1 cells that stably expressed Myc-AT2 receptor variant (Figure 4D). These results suggest the ligand-independent AT2-mediated growth inhibition is cell-type specific.

To probe for the underlying mechanism of the ligand-independent AT2-induced growth inhibition, cell cycle profile of stable cell clones was analyzed. As shown in Table 2, HEK293 and PC12 cells that stably expressed epitope-tagged AT2 receptors exhibited a partial cell cycle arrest. Regardless of the epitope tags, HEK293 cells expressing AT2 receptor variants showed a significant accumulation of cells in G1 phase (Table 2). By contrast, PC12 cells stably expressing Myc-AT2 receptor variant exhibited a significant increase of cell number in S phase (Table 2). Consistent with the proliferation rate analysis, no significant change in the cell cycle profile was observed in CHO cells that stably expressed either Myc- or GFP-tagged AT2 receptor variant (Table S3). These results suggested that ligand-independent AT2-mediated cellular responses were cell-type specific, but not affected by N- or C-terminal tagging.

### AT2 Receptors in Transiently Transfected Cells were Ubiquitinated

Unlike the detection of distinct high MW immunoreactive bands in stable cell clones, a ladder of immunoreactive Myc-AT2 proteins was detected in transiently transfected HEK293 cells (Figure 1C), indicating newly synthesized Myc-AT2 receptor variant was subject to a posttranslational modification other than glycosylation. Ubiquitination of GPCR has been reported to regulate GPCR sorting [30], [31], and hence a possibility arose that the ladder of immunoreactive protein bands could be resulted from ubiquitination of Myc-AT2 receptor variant. To investigate whether AT2 receptor is modified by ubiquitin, HA-tagged ubiquitin and N-terminally Myc-tagged or C-terminally GFP tagged AT2 receptor variants were transiently co-expressed in HEK293 cells. Pull-down analysis indicated that a series of HA-immunoreactive protein bands were detected in immunocomplexes precipitated with an anti-Myc antibody (Figure 5A and 5B) or an anti-GFP antibody (Figure 5C and 5D), suggesting that transiently expressed Myc-AT2 and AT2-GFP receptor variants were ubiquitinated. Unexpectedly, in stable transfected HEK293 cells that transiently over-expressed HA-ubiquitin, ubiquitination of stably expressed Myc-AT2 or AT2-GFP receptor variants was not detected in the absence or presence of ANGII (Figure 5A and 5B). These results suggest that ubiquitination of AT2 receptor is affected by mode of expression.

## Discussion

In the present study, four N-terminally or C-terminally tagged AT2 receptor variants were transiently or stably expressed in three cell lines representing different species and tissue origin. Results demonstrated that subcellular distributions, level of AT2 receptor expression and receptor-mediated cellular responses were cell type dependent. On the other hand, glycosylation of AT2 receptor were tag-dependent. Unexpectedly, AT2 receptor was ubiquitinylated in transient expression but not in stable expression.

### Effect of Cell Type on the Expression and Constitutive Activity of Angiotensin AT2 Receptor

The HEK293 and CHO-K1 cells had been widely used for AT2 receptor expression [32], [33], and over-expression of AT2 receptor in PC12 cells was also examined lately [34]. Previous studies indicated that PC12 cells less than 17 passages express AT2 receptor exclusively [35]. However, expression of AT2 receptor was not detected by RT-PCR in the wild-type PC12 cells used in the present study (data not shown). Similar to many other exogenously expressed GPCR [36], stably expressed AT2 receptor was mainly found on the cell membrane and in the cytosol of HEK293, CHO-K1 and PC12 cells. However, different cell lines showed different preference on subcellular distributions of the AT2 receptor (Table S2). The HEK293 cells provided the best membrane expression for all the epitope-tagged AT2 receptor variants in both transient and stable expressions. Although membrane expression was increased in stably transfected cells, the expression level of Myc-AT2 receptor variant was still low in CHO-K1 and PC12 cells (Figure 3, Table S2).

Cell proliferation assay and cell cycle profile analysis showed that the constitutive activity of AT2 receptor was dependent on cell type (Table 3). It is of interest to note that HEK293 and PC12 cells share similar activity profiles but being different from that of CHO-K1 cells. Variation of constitutive AT2 receptor activity in different host cells could be due to the differences of cellular proteome which engages the AT2 receptor to interact with different interacting partners, resulting in different cellular responses. Indeed, by using yeast two-hybrid system, it has been showed that the C-terminus of AT2 receptor interacts with the zinc finger protein PLZF from a human heart cDNA library [37] but with the Golgi protein ATBP50/ATIP from a mouse embryo cDNA library [38]. The PC12 cells were derived from adrenal medulla and HEK 293 cells were derived from kidney, in which endogenous expression of AT2 receptor could be detected even in adults [39], [40]. Thus, PC12 and HEK293 provide a more “native” environment, and therefore giving similar responses to AT2 receptor expression.

### Effect of Epitope-tag on AT2 Receptor Expression

Despite different epitope-tags are vastly different in size, and the intracellular C-terminal tail is important for GPCR signaling [41], confocal imaging study suggested that cellular distributions of C-terminally and N-terminally tagged AT2 receptor variants were not significantly different in HEK293 and CHO-K1 cells. However, immunoprecipitation study indicated that expression of AT2-FLAG receptor variant was substantially lower in both transient and stable expression. Furthermore, cell surface expression of Myc-AT2 receptor variant was facilitated by glycosylation, but was not required for AT2-GFP receptor variant. These results suggest epitope-tagging may exert a positive influence on receptor expression and sorting.

Molecular mass of AT2 receptor has been reported to be 40 kDa - 70 kDa in some cells and tissues [42], [43], [44], [45], [46], suggesting that the AT2 receptor is expressed as a monomer with different extends of glycosylation. However, in present study, various epitope-tagged AT2 receptor variants were found to form dimer and oligomer in spite of samples being prepared under reducing condition in the presence of β-mercaptoethonal, a reducing reagent. Consistent to our observations, it has been reported that some GPCR dimers were resistant to reducing reagent [47]. Recently, it has been reported that AT2 receptor expressed in PC12W cells forms dimer and oligomer, and homo-oligomerization of AT2-GFP receptor variant was detected in the membranes of stably transfected CHO-K1 cells [12]. Furthermore, AT2 receptor oligomers were also detected in brain tissues [48]. Importantly, those high molecular weight receptor oligomers were enriched in the membrane fraction, suggesting oligomerization of AT2 receptor plays a critical role in membrane localization.

### Effect of Transient and Stable Transfections on AT2 Receptor Expression

Immunofluorescent staining showed that stably transfected PC12 and CHO-K1 cells exhibited a greater extends of AT2 receptor cell surface expression than that of transient transfection. By contrast, the ratio of surface and cytosol expression in HEK293 cells was reduced in stably transfected cells (Table S2). In transient transfection, exogenous DNA does not integrate into genome and cause a high expression of transgene for a short period of time. In stable expression, exogenous DNA will be integrated into the genome and cause a sustained but relatively lower expression of transgene [49]. Hence, the differences between stably and transiently transfected cells on AT2 receptor cell surface expression suggest that high expression of AT2 receptor protein in transiently transfected cells may lead to receptor protein accumulation in ER and Golgi body, blocking the posttranslational modifications of AT2 receptor which is required for cell surface expression (see discussion blow).

Of interest, ubiquitination of AT2 receptor was only detected in cells that transiently expressed AT2 receptor. Consistent to our observation, AT2 receptor has been linked to an ubiquitin ligase MMS2 in mouse brain [50], [51]. Ubiquitination of transiently expressed AT2 receptor may be due to over-expression of AT2 receptor proteins. Over-expression of protein induces ER stress which in turn targets those partially fold proteins for ubiquitin-proteasome degradation [52]. On the other hand, ubiquitination of AT2 receptor may also have its physiological roles as tissue AT2 receptor expression is also “transient” in nature. In adult, AT2 expression is low or even undetectable. However, in wound healing and tissue injury, expression of AT2 receptor dramatically increases [9]. Hence, further work is required to ascertain the regulatory role of ubiquitination on the functions of AT2 receptor.

### Trafficking of Angiotensin AT2 Receptor

It is reported that AT2 receptor resided in cytosol in basal state. Following high concentration of ANGII treatment or serum starvation, AT2 receptor is translocated to cell membrane [12], [53]. It is commonly believed that glycosylation was not involved in AT2 receptor cell membrane expression [54]. Recently, a C-terminal di-acidic motif has been shown to regulate the trafficking of AT2 receptor between cell surface and endoplasmic reticulum [34]. Unlike other GPCRs, AT2 receptor is not internalized to cytosol after shot-term treatment with ANGII [55].

In the present study, it is noticeable that in the presence of tunicamycin, the homo-dimer and oligomer like immunoreactive protein bands were more prominent in cells expressing C-terminal tagged AT2 receptor variant (AT2-GFP and AT2-FLAG). By contrast, monomer like immunoreactive band was dominant in cells expressing N-terminal tagged Myc-AT2. Furthermore, it was found that cell surface expression of Myc-AT2, but not AT2-GFP, receptor variant was modified by glycosylation. It is unknown why glycosylation of AT2 receptor was affected by C-terminal GFP tagging. Importantly, glycosylation promoted the oligomer formation which in turn enhanced the cell surface expression of Myc-AT2 receptor variant. These results suggest that following synthesis in ER, AT2 receptor may undergo glycosylation and then forms homo-dimer or oligomer, which helps AT2 receptor travelling to the cell surface. Nascent and non-glycosylated AT2 receptor is subject to ubiquitination, which targets the receptor protein for degradation in proteasome. It is likely that newly synthesized and cell surface expressed AT2 receptors are in equilibrium, and signals such as serum starvation could enhance cell surface expression of AT2 receptor. When new equilibrium is established, the internalized AT2 receptor could be recycled (early-endosome) or degraded (proteasome or lysosome) like many other GPCRs [56].

### Heterologous Expression of GPCR

Expression of recombinant GPCR in heterologous cells is widely used to examine the receptor-mediated cell signaling pathways [57], to dissect the pharmacogenetic links to diseases [58], and to apply for receptor-based drug discovery [59]. Despite GPCR has been successfully expressed in various hosts including bacteria, yeast, insect and eukaryotic cells [60], results of present study clearly indicate that the host cells determine the levels of expression and the constitutive activity of receptor. Of interest, host cells that derived from tissues in which the GPCR expresses endogenously give similar responses, suggesting the essential of using homologous cell types for physiological characterization of GPCRs.

Epitope tagging facilitates the purification as well as the dynamic tracing of GPCR in tissues and cells [1], [61], in particular the specificity of the anti-GPCR antibody is in question [62]. Consistent with previous studies that epitope tag placed in the terminal ends or in the loop regions would not seriously affect the structure and functions of receptor protein [63], [64]. Unexpectedly, the terminal epitope tag was found to exert an influence on receptor protein expression and posttranslational processing. Furthermore, mode of expression (transient vs stable) was also found to modulate the receptor expression and posttranslational processing. Unfortunately it is difficult to predict how the terminal tag affects the receptor protein, and impacts of epitope on receptor protein might need to be determined experimentally.

Despite body of evidence indicate PD123319 is an AT2-specific non-peptide antagonist [65], [66], it is of interest to note that the proliferation rate of HEK293 cells was 30–50% higher in the presence of PD123319, regardless the HEK293 cells were stably expressing epitope-tagged AT2 receptor variant or transfected with empty vector (Figure 4b–c). The result suggests PD123319 may exert an AT2-independent growth-promoting effect in HEK293 cells. However, the nature of this growth-stimulating effect of PD123319 remains elusive.

In summary, results of present study suggest that subcellular distributions and receptor-mediated cellular responses of AT2 receptor are cell-type dependent. Moreover, N-terminal or C-terminal tagging does not significantly affect the expression and the ligand-independent activities of AT2 receptor. However, epitope tag and mode of expression may affect the expression levels and the post-translational processing of AT2 receptor. Of important, N-terminally Myc-tagged AT2 receptor exhibits typical GPCR features including glycosylation, oligomerization, membrane localization and ligand-independent constitutive receptor activities, indicating Myc-AT2 receptor variant is a better surrogate receptor for dissecting the signaling cascades and pathophysiology functions of AT2 receptor.

## Supporting Information

Figure S1 **Glycosylation on epitope-tagged AT2 receptor variants.** CHO cells that stably expressed AT2-GFP or Myc-AT2; PC12 cells that stably expressed Myc-AT2, and HEK293 cells that stably expressed AT2-FLAG (100 mm disk with >90% confluence) were treated with or without 1 µg/ml tunicamycin for 24 hr. Cells were then lysed in RIPA buffer, epitope-tagged AT2 is immunoprecipitated, and protein blot was probed with an anti-FLAG, an anti-Myc or an anti-GFP antibody as indicated. Specific immunoreactive protein bands are indicated with asterisks. Ab V_H_: Antibody heavy chain.
(TIF)

Table S1 **Summary of stable cell lines expressing epitope-tagged AT2 receptor variants.**
(DOCX)

Table S2 **Summary of AT2 receptor variant expressions in different cell types.**
(DOCX)

Table S3 **Cell cycle profile of stable CHO-K1 cell lines expressing epitope-tagged AT2 receptor variants.**
(DOCX)
