# Supplementary figures and images for: Impact of Cell Type and Epitope Tagging on Heterologous Expression of G Protein-Coupled Receptor: A Systematic Study on Angiotensin Type II Receptor
Source: PLoS One. 2012 Oct 8;7(10):e47016. doi: 10.1371/journal.pone.0047016 (PMC3466278; doi:10.1371/journal.pone.0047016)

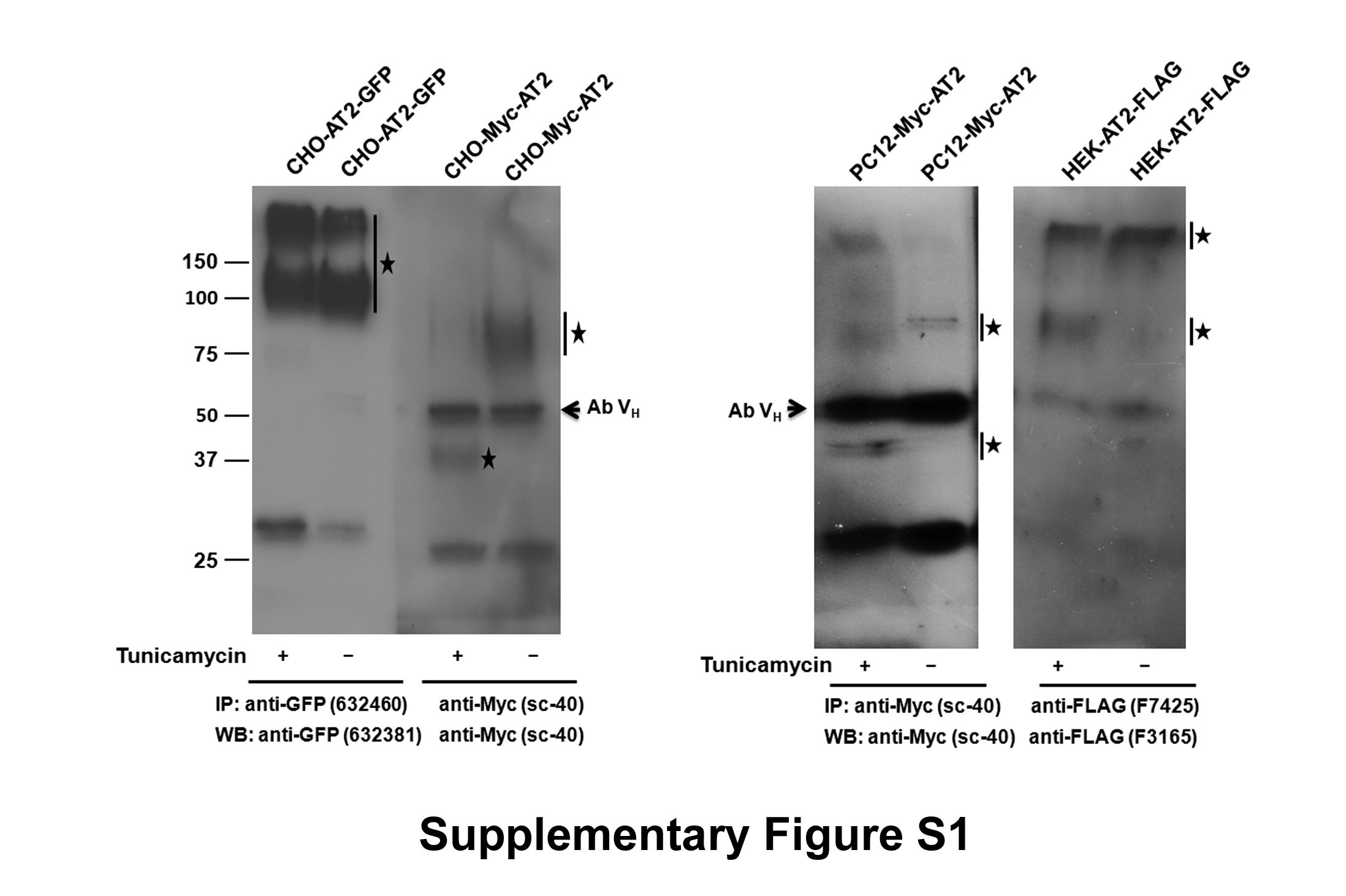

Supplement: Figure S1 — Glycosylation on epitope-tagged AT2 receptor variants. CHO cells that stably expressed AT2-GFP or Myc-AT2; PC12 cells that stably expressed Myc-AT2, and HEK293 cells that stably expressed AT2-FLAG (100 mm disk with >90% confluence) were treated with or without 1 µg/ml tunicamycin for 24 hr. Cells were then lysed in RIPA buffer, epitope-tagged AT2 is immunoprecipitated, and protein blot was probed with an anti-FLAG, an anti-Myc or an anti-GFP antibody as indicated. Specific immunoreactive protein bands are indicated with asterisks. Ab VH: Antibody heavy chain. (TIF) [file pone.0047016.s001.tif]
