# Supplementary material for: Impact of Cell Type and Epitope Tagging on Heterologous Expression of G Protein-Coupled Receptor: A Systematic Study on Angiotensin Type II Receptor
Source: PLoS One. 2012 Oct 8;7(10):e47016. doi: 10.1371/journal.pone.0047016 (PMC3466278; doi:10.1371/journal.pone.0047016)
Supplement: Table S1 — Summary of stable cell lines expressing epitope-tagged AT2 receptor variants. (DOCX) [file pone.0047016.s002.docx]

| **Epitope tag** | AT2-GFP | pEGFP-N1 | AT2-FLAG | pCMV-FLAG | Myc-AT2 | pCDNA3 |
| --- | --- | --- | --- | --- | --- | --- |
| **Cell line** |  |  |  |  |  |  |
| CHO-K1 | **CHO-AT2-GFP** | **CHO-GFP** |  |  | **CHO-Myc-AT2** | **CHO-PCDNA** |
|  | **C5 , C6** | **C11** |  |  | **C2, C4** | **C1, C2** |
| HEK 293 | **HEK-AT2-GFP** | **HEK-GFP** | **HEK-AT2-FLAG** | **HEK-FLAG** | **HEK-Myc-AT2** | **HEK-PCDNA** |
|  | **C1, C5** | **C6** | **C1, C4** | **C2, C4** | **C2, C3, C5** | **C5** |
| PC12 |  |  |  |  | **PC12-Myc-AT2** | **PC12-PCDNA** |
|  |  |  |  |  | **C2, C9, C12** | **C1,C2,C3** |

**Table S1: Summary of stable cell lines expressing epitope-tagged AT2 receptor variants.**

***** The clones in red colour gave the highest expression of epitope-tagged AT2 receptor variants and were used for subsequent characterization studies.
