# Supplementary material for: Impact of Cell Type and Epitope Tagging on Heterologous Expression of G Protein-Coupled Receptor: A Systematic Study on Angiotensin Type II Receptor
Source: PLoS One. 2012 Oct 8;7(10):e47016. doi: 10.1371/journal.pone.0047016 (PMC3466278; doi:10.1371/journal.pone.0047016)
Supplement: Table S2 — Summary of AT2 receptor variant expressions in different cell types. (DOCX) [file pone.0047016.s003.docx]

| **Cell Location** | | **HEK293** | **PC12** | **CHO-K1** |
| --- | --- | --- | --- | --- |
| **Transient** | **Cell surface** | **++++** | **−** | **+** |
|  | **Cytosol** | **++** | **++++** | **++++** |
| **Stable** | **Cell surface** | **+++** | **+** | **+++** |
|  | **Cytosol** | **++** | **++** | **+++** |

**Table S2: Summary of AT2 receptor variant expressions in different cell types.**

The + symbol represents the relative density of AT2 receptor variants at the sites indicated in the confocal images. The – symbol indicates no AT2 receptor variants is observed in the confocal image

\
