# Supplementary material for: Impact of Cell Type and Epitope Tagging on Heterologous Expression of G Protein-Coupled Receptor: A Systematic Study on Angiotensin Type II Receptor
Source: PLoS One. 2012 Oct 8;7(10):e47016. doi: 10.1371/journal.pone.0047016 (PMC3466278; doi:10.1371/journal.pone.0047016)
Supplement: Table S3 — Cell cycle profile of stable CHO-K1 cell lines expressing epitope-tagged AT2 receptor variants. (DOCX) [file pone.0047016.s004.docx]

**Table S3: Cell cycle profile of stable CHO-K1 cell lines expressing epitope-tagged AT2 receptor variants.**

|  | CHO-K1 | | | | |
| --- | --- | --- | --- | --- | --- |
|  | Myc-AT2 | |  | AT2-GFP | |
|  | Control | Experiment |  | Control | Experiment |
| G1 | 44.83 ± 4.69 | 43.23 ± 4.95 |  | 46.43 ± 2.48 | 51.84 ± 6.72 |
| S | 53.18 ± 3.88 | 49.33 ± 6.95 |  | 50.82 ± 2.48 | 47.21 ± 6.56 |
| G2/M | 2.00 ± 0.96 | 7.44 ± 2.07 |  | 2.75 ± 0.52 | 0.71 ± 0.48 |

Stably transfected CHO-K1 cells (2×10^5^ cells) were seeded in 60 mm dishes. After 2 days, the cells were collected and cell cycle analysis was performed as described in Methods. Cells stably transfected with empty vector were used as control. Distributions of cells in each phase of a cell cycle were expressed as percentage of total cell analyzed. Data shown is mean ± SEM of 4 independent experiments. Differences between means were compared with unpaired Student’s *t*-test using GraphPad Prism 5. No statistical significant different was detected between means.
